# Coronavirus Disease (COVID-19); Lessons Learnt from International Response and Advice to the Georgia Government

**DOI:** 10.1016/j.xinn.2020.100025

**Published:** 2020-07-31

**Authors:** Minaz Mawani, Changwei Li

**Affiliations:** 1 Department of Epidemiology and Biostatistics, College of Public Health, University of Georgia, Athens 30606, USA; 2 Department of Epidemiology, Tulane University School of Public Health and Tropical Medicine, 1440 Canal Street Suite 2000, New Orleans, LA 70112, USA

## Abstract

This commentary presents an analysis of the containment and mitigation efforts by different countries against the recent COVID-19 pandemic. It was developed in response to the Georgia government's decision to relieve lock down restrictions. The article also provides recommendations based on interventions that have been observed to be effective, which will guide decision making for not only Georgia but other states and countries that are currently struggling to manage this outbreak.

## Main Text

**Figure 1:**
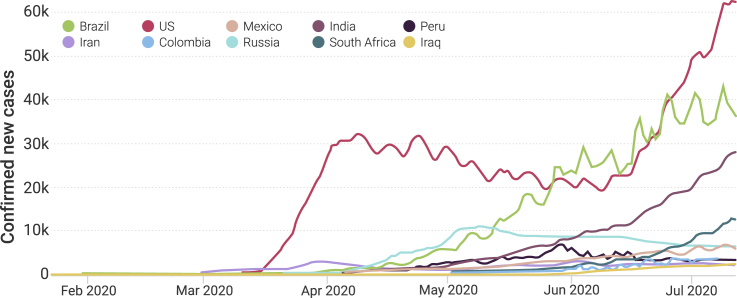
Five-Day Moving Average Showing Daily Confirmed New Cases for the Ten Most Affected Countries

In December 2019, a new coronavirus (SARS-CoV-2) was identified as the causative agent of a severe acute respiratory disease (COVID-19) in Wuhan, China, with cases being linked to a wholesale seafood market selling a variety of live animals.^1^ This was soon declared a pandemic by the World Health Organization.^1^ Currently more than 13 million confirmed cases and more than 500,000 deaths have been reported around 188 countries globally. The United States is now considered to have widespread community transmission with more than 3 million cases and 135,000 deaths.^2^ Figure 1 shows the trends of daily confirmed new cases across the ten most affected countries. The top three countries reporting the highest number of cases are the United States, Brazil, and India.^2^

The virus has some similarities with past SARS (2002–2003) and MERS (2012 to ongoing) outbreaks, both of which had zoonotic transmission and a similar symptom profile. However, COVID-19 is comparatively more contagious and associated with poor outcomes such as hospitalization, ICU admission, and death. Therefore, urgent steps are required to control and mitigate the disease.^3^ The strategies that have worked in past to control SARS and MERS may not be efficient to control COVID because it differs in terms of infectious period, transmissibility, clinical severity, and extent of community spread,^3^ with still some uncertainties about pathophysiology and transmission. Globally, besides affecting the physical health of individuals, it has led to psychological impacts due to confinement and fear, disruption of the economy and supply chains, and exhaustion of health care resources.^1^^,^^4^

Different countries and regions have had different responses to the spread, which is worth a discussion. Italy could not act in a timely manner and started issuing decrees that gradually increased restriction with lockdown until it applied to the whole country, but by this time they were already experiencing a serious health care resource crisis, which led to rationing of life-support interventions to those who were most likely to survive.^4^

Countries such as New Zealand, China, and Singapore have been able to respond effectively and contained the virus.

On May 3, 2020, New Zealand reported its first day of having no new cases of Covid-19. A month after they recorded their first case, they had committed to an elimination strategy and a strict national lockdown was announced by their government. This included implementing a full lockdown, involving the closure of schools and non-essential workplaces, a ban on social gatherings, and severe travel restrictions. These steps allowed them to do effective contact tracing, border control, and surveillance. Their official response was guided by the principle of not stigmatizing and instead being united against eliminating the disease.^5^

China took drastic measures to restrain any mass gatherings, including Chinese new year celebrations, isolation and quarantine of cases and contacts, community containment, social distancing, mandatory use of facemasks for all, a lockdown of transportation at national level, and construction of two new hospitals over two weeks to manage COVID-19 patients.^1^ Taiwan, a part of China, leveraged their health insurance database with immigration and customs for creation of big data. In addition, generating real-time alerts to identify and track cases through mobile phones, government support for food and supplies for people under quarantine, border control, public education, using government funds to produce more personal protective equipment (PPE) along with setting prices for those were some of their initiatives to control the disease.^6^

Singapore responded even before COVID-19 was identified as a disease. Beginning in January, temperature screening was initiated at the airports. Other measures included aggressive contact tracing, isolation and quarantine of close contacts, travel advisories, and entry restriction. This country had strengthened their systems to deal with another outbreak after SARS in 2003. They have built a national center for infectious diseases with negative pressure isolation rooms along with stocking of PPE, training health care professionals, communication with the public, and active engagement of frontline workers.^7^

United States has been particularly slow to respond with varying levels of restrictions implemented throughout the states. The state of Georgia initially had a delayed response where a public health emergency was announced on March 14, however, a shelter in place order was implemented on April 2nd and the restrictions were later relaxed from May 1 to 13. The recent guidelines are mostly targeted at symptomatic or high-risk individuals despite the fact that asymptomatic carriers can spread the disease.^8^ Since the number of cases and deaths have been increasing despite all interventions, relieving restrictions at this point might not be a good idea. In fact, there is a need to move from containment to mitigation efforts. In this regard, the following are some of the recommendations that we would like to make.

For symptomatic and exposed individuals and their close contacts, we recommend rigorous tracing and quarantine. We also recommend improving surveillance systems, especially at all entry and exit points, investing in big databases through developing linkages for reporting and monitoring of symptoms.

For protecting unexposed, at individual level, we recommend staying at home unless necessary, practicing contact precautions, social distancing, and mandatory use of facemasks when stepping outside.

At city level, we recommend extending shelter in place orders with strict implementation of social distancing measures that include restricting movement in all public places and transportation.

At state level, we recommend developing mechanisms to protect vulnerable individuals such as older adults, people with chronic diseases, and those with poor immune systems; increasing intersectoral collaboration, local production of medical supplies and PPE, effective communication between governments, health care authorities, and the local population; developing health and hygiene protocols to limit live animal and human contact, surveillance of animal markets, and thoroughly cooking eggs and meat; investing in proper awareness programs targeting the general population through easy to follow uniform messages using different media sources and dealing with myths and misinformation in a timely and effective manner.

At the level of health care systems, we recommend increasing the budget allocation for health care and research; ensuring availability of PPE, avoiding hospital overcrowding, and using telehealth systems to protect frontline staff from exposure; investing in developing specialized hospitals with trained staff and negative pressure isolation rooms to manage infectious disease outbreaks. All these interventions will help slow down the spread and would buy time for health systems to scale up and respond until vaccines and treatments are available.

Lastly, there needs to be a well thought plan for dealing with future outbreaks, focusing not just on disease control but also alternative ways of carrying out day to day tasks safely.^1^, ^2^, ^6^, ^7^, ^8^, ^9^, ^10^
